# Selection in spatial working memory is independent of perceptual selective attention, but they interact in a shared spatial priority map

**DOI:** 10.3758/s13414-015-0976-4

**Published:** 2015-09-04

**Authors:** Craig Hedge, Klaus Oberauer, Ute Leonards

**Affiliations:** School of Psychology, Cardiff University, Tower Build, Park Place, Cardiff, CF10 3AT UK; Department of Psychology, University of Zurich, Zurich, Switzerland; School of Experimental Psychology, University of Bristol, Bristol, UK

**Keywords:** Working memory, Attention, Attention switching, Focus of attention, Spatial attention

## Abstract

We examined the relationship between the attentional selection of perceptual information and of information in working memory (WM) through four experiments, using a spatial WM-updating task. Participants remembered the locations of two objects in a matrix and worked through a sequence of updating operations, each mentally shifting one dot to a new location according to an arrow cue. Repeatedly updating the same object in two successive steps is typically faster than switching to the other object; this object switch cost reflects the shifting of attention in WM. In Experiment 1, the arrows were presented in random peripheral locations, drawing perceptual attention away from the selected object in WM. This manipulation did not eliminate the object switch cost, indicating that the mechanisms of perceptual selection do not underlie selection in WM. Experiments 2a and 2b corroborated the independence of selection observed in Experiment 1, but showed a benefit to reaction times when the placement of the arrow cue was aligned with the locations of relevant objects in WM. Experiment 2c showed that the same benefit also occurs when participants are not able to mark an updating location through eye fixations. Together, these data can be accounted for by a framework in which perceptual selection and selection in WM are separate mechanisms that interact through a shared spatial priority map.

One of the key debates about the structure and functioning of working memory (WM) concerns the degree to which the mechanisms underlying perceptual attention play a role in WM processes, and how WM contents modulate perceptual-attention processes (for reviews, see Awh, Vogel, & Oh, 2006; Gazzaley & Nobre, 2012; Olivers & Eimer, 2011; Olivers, Peters, Houtkamp, & Roelfsema, 2011; Theeuwes, Belopolsky, & Olivers, 2009). In this article, we contribute to this debate by investigating the relation between perceptual attention and attention to the contents of WM. Both forms of attention serve to select some representations (often just one) over others. Whereas perceptual attention is directed externally, toward stimuli, objects, events, or spatial locations in the perceived environment, attention in WM is directed internally, to select a representation from the current contents of WM. Accordingly, some authors have referred to the foregrounded state of a single item in WM as the *focus of attention* (FoA; for a review, see Oberauer & Hein, 2012; see also the work of McElree and colleagues: McElree, 2001, 2006; McElree & Dosher, 1989, 1993; Wickelgren, Corbett, & Dosher, 1980). In these models of WM, the FoA refers to the privileged access to cognitive operations for (typically) a single item or chunk of information.

The different states of representation in WM make a difference regarding how WM interacts with perceptual attention, since recent evidence has suggested that the FoA in WM shares a unique relationship with perceptual processing. When one is performing a visual search task, an item held in WM automatically biases attention toward matching targets or distractors in the display (Olivers & Eimer, 2011; Olivers et al., 2011). This bias was found to be restricted to a single item, and therefore, Olivers and colleagues proposed a distinction between “passive” memory items and a single-item “attentional set” in WM (e.g., a target to be searched for), which can bias the processing of perceptual inputs. The notion that the FoA in WM uniquely biases perceptual processes raises a question about the reverse direction of causality: To what extent are the mechanisms of perceptual attention involved in creating and maintaining the FoA in WM? The experiments described here were designed to address the interaction between attention deployed to internal representations and to external stimuli by manipulating both factors independently.

## The relationship between perceptual attention and WM

The work of Awh and colleagues provides prominent evidence that the mechanisms underlying perceptual attention also modulate the status of items in WM (Awh & Jonides, 1998; Awh, Jonides, & Reuter-Lorenz, 1998; Awh et al., 2006). They reported that shifting perceptual attention away from the location of a memorized item to perform a perceptual discrimination task reduced the accuracy of memory for the item’s location, though a discrimination task not requiring a spatial shift of attention did not (but see Belopolsky & Theeuwes, 2009b; Chan, Hayward, & Theeuwes, 2009). Furthermore, there is evidence that spatial attention plays a role in the retrieval of items from memory (Theeuwes, Kramer, & Irwin, 2011). Throughout this article, we use the term “perceptual attention” to refer to what Awh and colleagues (e.g., Awh et al., 1998) called “spatial selective attention”—that is, the mechanisms underlying the selection, and subsequent discrimination, of a stimulus in a visual scene.

There is, however, also evidence for some degree of independence between the prioritization of an item in WM and perceptual attention: The selective retention of a prioritized item in WM is robust to shifts of visual attention during a probed-recall task (Hollingworth & Maxcey-Richard, 2013; Maxcey-Richard & Hollingworth, 2013; Rerko, Souza, & Oberauer, 2014). Maxcey-Richard and Hollingworth noted the apparent contradiction of these findings with those of Awh et al. (1998), suggesting that memory for spatial location (as measured by Awh et al., 1998) may draw upon different mechanisms than memory for properties such as color, which their task probed. Additionally, these studies primarily examined the impact of perceptual attention shifts on maintaining an item in the FoA, rather than the selection of items into the FoA. In contrast, here we manipulated shifts of both the FoA in WM and perceptual attention in a single-task framework.

### The focus of attention in WM

One experimental paradigm for studying the FoA in WM is the object switch paradigm: Garavan (1998) had participants keep a running count of two categories of shapes through a sequence of additions (e.g., “add one to the squares count”). Participants were required to press a single response button as quickly as possible when they had completed each update, and their reaction time (RT) from the onset of a shape to the buttonpress was recorded. Garavan observed longer RTs when participants were required to update different counts consecutively (e.g., incrementing the squares count after incrementing the circles count), as compared to when they incremented the same count across two trials. Garavan interpreted this switch cost as the time it takes for the FoA to switch from one object in WM to another. Note that this RT difference may be framed better as a repetition benefit for an operation performed on the item currently in the FoA. Subsequently, similar switch costs/repetition benefits were observed in arithmetic-updating (Oberauer, 2002, 2003) and spatial-updating tasks (Kubler, Murphy, Kaufman, Stein, & Garavan, 2003; Oberauer & Bialkova, 2009).

**Fig. 1:**
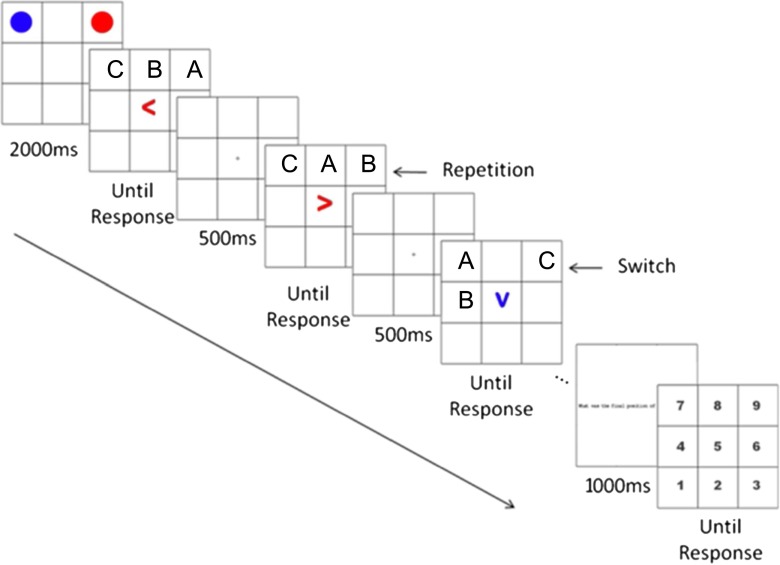
Diagram of a trial sequence from the task used in Hedge and Leonards (2013). Participants are to mentally update the location of the object indicated by the color of the arrow one square in the direction of the arrow, pressing a single response button to indicate completion of the update. For example, a red leftward-pointing arrow was a cue to update the red circle one square to the left from its location in WM. Participants indicated the final positions of both objects at the end of each sequence by pressing a corresponding numbered key. In one version of the task, eye movements were unconstrained; in another, the appearance of each arrow cue was contingent upon refixating the center between updates. The letters (not seen by the participant) reflect the locations defined for our analysis: (A) the old location of the object currently being updated; (B) the new location of the object currently being updated; (C) the location of the object not currently being updated, referred to as the “passive location.” From “Using Eye Movements to Explore Switch Costs in Working Memory,” by C. Hedge & U. Leonards, 2013, *Journal of Vision*, *13*(4), 18. Copyright 2013 by ARVO. Adapted with permission

We have previously used a spatial variant of the object-switching task to examine the overlap between the selection of information in WM and perceptual selection (Hedge & Leonards, 2013). We used a modified version of this same paradigm in the present experiments; thus, we shall describe the design in detail. In this task, participants were required to mentally update the locations of two circles in a 3 × 3 grid, while concurrently having their eye movements recorded. Participants worked through a series of updating steps, each of which consisted in shifting one or the other circle to an adjacent grid location. The direction of each updating step was indicated by a centrally presented colored arrow; the arrow’s color determined which circle the update was to be applied to. The main dependent variable was the RT for each update, measured from the onset of an arrow until participants pressed the space bar to continue to the next update. A schematic of this task is shown in Fig. 1.

Performing each update entails at least three steps. First, participants must identify the color of the target object and the instructed direction of the update by processing the arrow. Second, they must select the correct object in WM. Third, they must compute the new location of the object while maintaining the other object in WM at its unchanged location.

In Hedge and Leonards (2013), we observed that eye movement patterns corresponded closely to the location of the object currently being updated in WM and were predictive of the RTs for each update. Updates on which participants’ first saccade was directed toward the object currently being updated were associated with faster RTs, suggesting that the allocation of perceptual attention corresponds to the allocation of priority in WM.

We accounted for these findings within a framework in which WM and perceptual attention share a *spatial priority map* (Belopolsky & Theeuwes, 2009a; Theeuwes et al., 2009), in which the activity of locations reflects the degree of attention allocated both to the items held in WM and to perceptual stimuli. Activation in this map feeds into the eye movement system, causing eye movements to be directed toward items with high relative priority in this map. Theeuwes et al. speculated that sustained activity in this map could also underlie the maintenance of items in WM, similar to other accounts of the relationship between attention and WM (e.g., Postle, 2006). As an extension of this idea, it could be that the benefits associated with the FoA are a manifestation of *relative* differences in activity in this map.

The correspondence between eye movements and updating latencies that we observed previously does not directly address the functional relationship between perceptual attention and the FoA. Three possible interpretations could explain these findings: (i) The FoA in WM is dependent on perceptual attention, so that they must always be aligned in space; (ii) the FoA in WM is completely independent from perceptual attention, and the eye movements we observed were epiphenomenal; and (iii) selection and prioritization of an item in WM is independent from what is selected by perceptual attention, but the two forms of attention interact in a shared representation of spatial priority.

In the present article, we adapted the spatial-updating task to independently manipulate shifts of perceptual attention and the locations of objects in WM, allowing us to test the predictions of these three hypotheses. If the mechanisms underlying perceptual attention also support priority in WM, then manipulating perceptual attention shifts should directly impact upon the switch cost/repetition benefit.

### Task and predictions

**Fig. 2:**
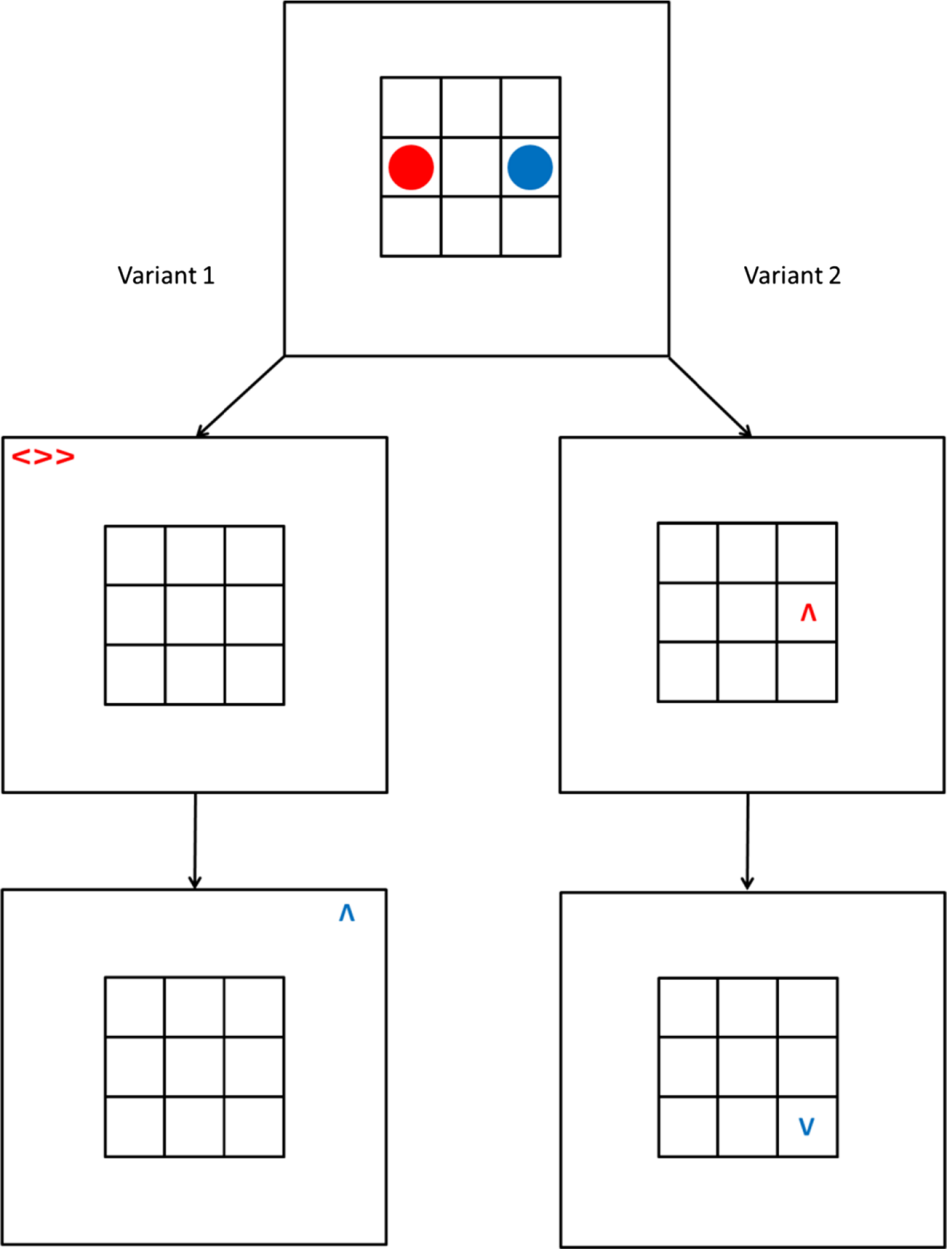
Schematic of two variants of the spatial-updating task. In Variant 1 (left side), the arrow cues were presented outside the grid, such that perceptual attention is always drawn away from the location of the object to be updated. In the three-arrow version (illustrated in the first update), two of these arrows face in one direction, with the remaining arrow facing in the opposite direction. Participants were required to determine the dominant direction of the arrows in the cue and to use this direction for their WM update. The single-arrow version (shown in the lower left frame) consisted of a single arrow cue presented in a peripheral location. In Variant 2 of the task (right side), arrow cues were presented within the grid, and could overlap with either the old or the new (as shown in the second update) location of the object to be updated in WM, or could be presented in the location of the passive memory item (as in the first update), or could be in another location, not currently occupied by either object. In Experiment 2a, we used a smaller grid size (2 × 2) than we had previously used. Experiment 2b replicated the design using the 3 × 3 grid, and Experiment 2c replicated Experiment 2b while additionally controlling for participants’ use of eye movement strategies

To derive predictions for the present study, we adopted a logic similar to that of Awh et al. (1998); that is, if perceptual attention underlies the prioritized state of the FoA in WM, we should observe a reduction or elimination of the repetition benefit when perceptual attention is drawn away from the memory item in the FoA. In contrast, we should observe faster RTs when perceptual attention and the FoA in WM are aligned. To incorporate this logic into our paradigm, we manipulated the location of the arrow cue indicating the update direction, and used the arrow location to control which location is selected by perceptual attention. We created two variants of the task illustrated in Fig. 1. In the first variant (Exp. 1), the arrow cue always appeared in one of four random locations in the periphery (i.e., outside the grid containing the to-be-updated objects). We used two kinds of arrow cues: single arrow heads and a group of three arrow heads. We assumed that the finer perceptual discrimination required by the three-arrow cue would mirror the requirement for spatial selective attention in discriminating an item from peripheral distractors in the Eriksen flanker task (Eriksen & Eriksen, 1974), thus ensuring that participants were required to shift their perceptual attention away from the location of the current FoA in WM. In the second variant of our paradigm (Exps. 2a, 2b, and 2c—see the Fig. 2 caption for details), the arrow cues were presented in different locations within the grid, allowing us to compare the RTs on updates in which the arrow cue was aligned with the object to be updated or was in a different location. The two variants of the paradigm are illustrated in Fig. 2.

**Fig. 3:**
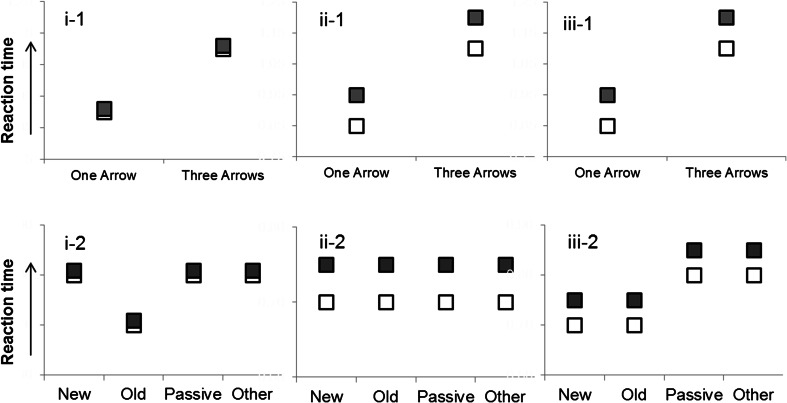
The patterns of data predicted by each of our three hypotheses—(i) the dependence hypothesis, (ii) the independence hypothesis, and (iii) the shared-map hypothesis—for Experiment 1 (top row) and Experiments 2a, 2b, and 2c (bottom row). The typically observed object switch cost/repetition benefit reflects faster reaction times in repetition updates (white symbols) than in switch updates (gray symbols). See the text for further details

We tested the three hypotheses raised above, investigating the relation between perceptual attention and attention in WM; their predictions with regard to the RT patterns are illustrated in Fig. 3.

#### (i) The FoA in WM is dependent on perceptual–spatial attention

The dependence hypothesis states that the selection of a location in WM depends on the selection of that location through the same mechanisms that underlie perceptual selection. Hence, when the arrow cue is presented in a different location from the object to be updated, the RT repetition benefit should be abolished.^1^ In Experiment 1 (arrow cue spatially separated from the test grid), we should observe no repetition benefit/switch cost in RTs, since perceptual attention is always drawn away from the object held in the FoA. Our predictions would be the same for both the single- and three-arrow conditions, though the latter would act as a stronger test by ensuring that participants needed to shift to the location and determine the update operation in the presence of competing information in close proximity. In Experiments 2a–2c (arrow cue location varied within the test grid), one would predict an RT benefit in the case of the arrow cue appearing in the old location of the object to be updated, as compared to trials on which the arrow cue appeared in any other location. These predictions are shown in panels i-1 and i-2 of Fig. 3.

#### (ii) The FoA in WM is independent of perceptual–spatial attention

According to this hypothesis, perceptual–spatial attention plays no role in maintaining the FoA’s prioritized state. We should thus observe the typical RT switch cost in Experiment 1, and no effect of the arrow location manipulation in Experiments 2a–2c. See Fig. 3, panels ii-1 and ii-2.

#### (iii) The FoA and spatial attention are distinct mechanisms, but they interact through a shared spatial map

This scenario draws on the account offered in our previous work, as well as the frameworks proposed by Theeuwes et al. (2009). We assumed that perceptual–spatial attention and the FoA in WM have separate foci that can be oriented independently to different locations, but that they communicate through a shared priority map. We also assumed that decision time is influenced by the extent of competition in the map, with a more distributed pattern of activity leading to longer RTs. These considerations imply that spatial perceptual selection and selection in WM should have independent, additive effects on RTs. Yet, the processing of a perceptual stimulus or a representation in WM should be facilitated when the two sources of activity in the priority map are aligned.^2^

In Experiment 1 (arrow cue spatially separated from the test grid), we should observe the typical object switch cost in RTs. In Experiments 2a–2c (arrow cue location varied within the test grid), in addition to the object switch cost, we expected updating steps to be faster when the location of the arrow cue overlapped with the object to be updated, because directing perceptual attention to those locations facilitates selection of the same location in WM. Attentional overlap in the old location would facilitate orienting the FoA to that location as a starting point for the computation of the new location. Attentional overlap in the new location would facilitate computing the new location and shifting the FoA to it. Note that, in contrast, the dependence hypothesis (i) predicts a benefit for an arrow in the old location but not for an arrow presented in the new location, since it entails that only a single location can be prioritized for both perceptual processing and WM. Participants would need to process the arrow before determining the target of the update, so that an arrow in the new location would force them to initially switch attention away from the object itself. See Fig. 3, panels iii-1 and iii-2.

## Experiment 1: does priority in spatial WM require perceptual–spatial attention?

The first experiment modified the paradigm used in our previous work (Hedge & Leonards, 2013) to draw perceptual attention away from the current FoA in WM. In the previous version of the task, the arrow indicating the target and direction of the update always appeared in the center of the grid containing the two objects. In our present version, the arrows were presented in peripheral locations (i.e., outside the grid), appearing at random in one of the four corners of the screen. We reasoned that, if the FoA in WM is dependent on perceptual attention, the requirement to shift perceptual attention to a random location in order to discriminate the arrow cue should eliminate or reduce the typically observed RT repetition benefit. In other words, effectively every update would become a switch update.

### Method

#### Participants

Groups of 14 (single-cue version) and 16 (three-cue version) undergraduate students, 18–35 years of age, took part in the study for course credit. The participants in all experiments had normal or corrected-to-normal vision. All participants gave their informed written consent prior to participation, in accordance with the revised Declaration of Helsinki (2013), and the experiments were approved by the local Ethics Committee.

#### Design and procedure

Stimulus presentation and data recording were conducted using MATLAB 2008b with Psychophysics Toolbox 3.0.8 (Brainard, 1997; Pelli, 1997), presented on an 18-in. monitor with a 1,280 × 1,024 resolution. Participants were required to mentally update the positions of two objects in a 3 × 3 grid through a sequence of mental shifts. At the participant’s viewing distance of 57 cm, the black-lined grid subtended a visual angle of 11.22° horizontally and vertically on a mid-gray background (28.2 cd/m^2^). The two objects, presented only at the beginning of each trial in the grid square of their initial position, were 2.52° in diameter. The objects were a red circle (CIE *x* = .472, *y* = .334, *L* = 37.5 cd/m^2^) and a blue circle (CIE *x* = .177, *y* = .190, *L* = 37.5 cd/m^2^). Each update consisted of moving one of the circles by one square in a direction indicated by a colored arrow (0.56° in height and width). The color of the arrow indicated the object that was to be updated. The directions of movement were selected at random, with the constraints that the shifted circle stayed within the grid and did not occupy the same position as the other circle. The sequence of update types (repetition or switch) was pseudorandomly determined, with each trial type being equiprobable across the session. These features ensured that the cue location and update type were unpredictable to participants.

In the single-cue condition, a single arrow cue appeared randomly in one of four locations outside the grid, 6° outside the external corners (12° from the center of the screen). In the three-cue condition, the middle of three parallel arrows was presented in one of the same locations as in the single-cue condition, and was flanked by two arrows 0.76° away. One of the arrows always mismatched the other two, and the relative locations of the two matching and one nonmatching arrow cues within this arrow trio were randomly determined on each update. The dominant direction in the three-arrow group indicated the direction of the WM update. The first update in each sequence, which could not be categorized as a repeat or switch, was excluded from the analysis. At the end of each trial, participants were required to indicate the final positions of both circles. A trial was counted as correct only if both final circle positions were identified correctly.

A single trial consisted of the presentation of the grid with the starting positions of the red and blue circles for 2,000 ms, which was then replaced by a blank grid for 500 ms. This was followed by an arrow indicating the target and direction for the updating step, which remained on screen until the participant had pressed the mouse button to indicate that the required mental shift of the object was completed. The RT for each update was measured as the time from the onset of the arrow until the mouse button response. After the buttonpress, the blank grid was presented for 500 ms prior to presentation of the next arrow cue. A schematic of the three-arrow update cue can be seen in Fig. 2; otherwise, the trial sequence progressed in a form similar to that illustrated in Fig. 1.

Trial sequences consisted either of five, six, or seven updating steps, to discourage anticipation of the end of the sequence. After the last updating step, participants were prompted by a red question mark to indicate the final position of the red circle. Participants reported the current position of the red circle by clicking on the appropriate square. The final position of the blue circle was then probed in the same manner. Participants completed 12 blocks of 15 trials each, preceded by one practice block of five trials. Participants were instructed to respond to each updating step as quickly as possible, but not until they had completed the update. Only updates from trial sequences in which participants correctly identified the final locations of both objects were included in the analysis. Note that we were not interested in the accuracy of recall per se, and the task was not designed to elicit errors. The memory probes were included to ensure that participants were engaging in the task.

### Results and discussion

One participant’s data from the three-cue condition were removed from the analysis due to low performance (more than 3 *SD*s below the group average).

#### Task performance

On average, participants were correct on 79.84 % (*SD* = 13.14) of trials in the single-cue task, and on 84.67 % (*SD* = 11.09) in the three-cue version. A mixed 2 (cue number) × 3 (sequence length) analysis of variance (ANOVA) showed no significant effect of cue number, *F*(1, 27) = 1.147, *MSE* = 158.746, *p* = .29. A significant effect was shown for trial length, *F*(2, 54) = 5.078, *MSE* = 8.863, *p* = .01. Also, the planned linear contrast of sequence length showed a significant negative trend in memory performance, *F*(1, 27) = 7.245, *p* = .012. The interaction between cue number and sequence length did not reach significance, *F*(2, 54) = 0.596, *MSE* = 8.863, *p* = .555. Thus, the additional processing required to discriminate the three cues did not lead to a decrease in updating accuracy.

#### Reaction times

**Fig. 4:**
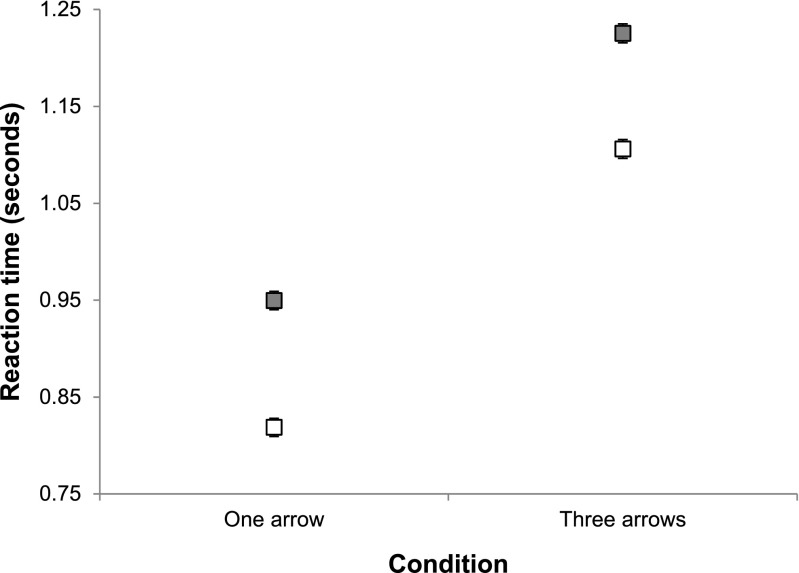
Average median reaction times for repetition (white) and switch (gray) updates for the single-arrow (*N* = 14; left) and three-arrow (*N* = 15; right) versions of the task in Experiment 1. Error bars indicate ±1 *SEM* corrected for within-subjects comparisons (Bakeman & McArthur, 1996)

Participants’ median RTs were analyzed with a mixed 2 (cue number) × 2 (object switch condition) ANOVA. A significant effect was observed for cue number, *F*(1, 27) = 9.421, *MSE* = 0.122, *p* = .005. Participants were faster on average in the single-cue condition (*M* = 884 ms, *SD* = 198 ms) than in the three-cue condition (*M* = 1,166 ms, *SD* = 295 ms), indicating that, as intended, the three-arrow cue was substantially harder to process than the single-arrow cue. That increase in difficulty arose from the additional selection demand in the three-cue stimuli, implying a higher demand on perceptual attention by the three-arrow cue. Critically, the effect of switch condition was significant, *F*(1, 27) = 85.583, *MSE* = 0.03, *p* < .001, with participants performing repetition updates (*M* = 967 ms, *SD* = 265 ms) faster than switch updates (*M* = 1,092 ms, *SD* = 301 ms), despite the requirement to spatially shift perceptual attention from the location of the current FoA in WM between updates. Furthermore, the interaction between cue number and update type did not reach significance, *F*(1, 27) = 0.183, *MSE* = 0.003, *p* = .672, indicating that the additional attentional demand for discriminating the three-arrow cues did not impact on the object switch cost. As can be seen in Fig. 4, the magnitudes of the switch cost were very similar in the single-arrow (131 ms) and three-arrow (119 ms) conditions, and were of a similar magnitude to that observed in our previous work (162 ms; Hedge & Leonards, 2013).

The findings from Experiment 1 indicate that simply drawing perceptual attention away from the location of the current FoA in WM does not remove the last-updated item from its privileged position in WM, even when perceptual attention must be used to discriminate between competing elements. In other words, the mechanisms responsible for selective attention in perception do not underlie selective attention in WM, in contrast to the dependence hypothesis (see Fig. 3, panel i-1).

This conclusion does not contradict our previous conclusion (Hedge & Leonards, 2013) that perceptual attention shifts closely correspond to priority in WM. However, it does prompt the need for a clarification of this relationship. In Experiments 2a, 2b, and 2c, we examined whether perceptual attention and the FoA in WM are completely independent, or whether there is a benefit when the target of perceptual attention is aligned with the current FoA in WM, relative to when it is not, contrasting the predictions of the independence (ii) and shared-map (iii) hypotheses.

## Experiment 2: is there a benefit for aligning the target of perceptual attention and attention to objects in WM?

In Experiment 1, we examined whether the switch cost would be eliminated by the requirement to shift attention with every update, and observed that a robust switch cost was still present. This was predicted by both the independence (ii) and shared-map (iii) hypotheses above, whereas the dependence hypothesis (i) predicted a reduced or absent switch cost/repetition benefit, and thus could already be discarded. Discerning between the two remaining accounts required a comparison between updates in which participants shifted perceptual attention away from the location of the current FoA in WM and updates in which they did not, which we addressed in the next experiment.

In the original version of our task (Hedge & Leonards, 2013), the arrow cue was always presented in the center of the update grid. Objects sometimes did and sometimes did not fall in this center square. However, because the location of the cue was constant, and thus very predictable, the alignment of memory objects with the cue location was confounded with the location of the memory objects (i.e., aligned objects must have been in the center of the grid). In Experiments 2a and 2b, we modified the task so that the arrows indicating the update appeared within the grid, but were presented in different squares within the grid in an unpredictable way. Whereas our original task had used a 3 × 3 grid, we reduced the grid to a 2 × 2 grid in Experiment 2a, in order to obtain enough trials per design cell in a single session. In Experiment 2b, we replicated the method with the 3 × 3 grid for consistency with earlier work.

The locations within the grid had different relevances to the objects on a given update, which we define below (cf. Hedge & Leonards, 2013), along with their frequency of occurrence.

- *Old*: The previous position of the to-be-moved object (Exp. 2a, 24.7 % probability; Exp. 2b, 11.3 %; Exp. 2c, 10.9 %)
- *New*: The new (updated) position of the to-be-moved object (Exp. 2a, 24.2 %; Exp. 2b, 11.3 %; Exp. 2c, 10.9 %)
- *Passive*: The position of the unmoved object (Exp. 2a, 24.9 %; Exp. 2b, 11.1 %; Exp. 2c, 11.3 %)
- *Other*: Any other square in the grid (Exp. 2a, 26.2 %; Exp. 2b, 66.4 %; Exp. 2c, 67 %)

To reiterate, the independence hypothesis (ii above) predicts a main effect of switching and no effect of arrow location on the RTs. The shared-map hypothesis (iii above) predicts main effects of both location and switching; there should be a beneficial effect on RTs when the locus of perceptual attention matches the old or the new location of the to-be-updated object, and this effect should be additive with the object switch effect.

## Experiment 2a

### Method

#### Participants

Fifteen undergraduate and postgraduate students, 18–33 years of age, took part in the study for course credit.

#### Design and procedure

The general design and procedure were the same as in Experiment 1, with the following exceptions. The objects were presented in a 2 × 2 grid, subtending a visual angle of 11.22° horizontally and vertically on a mid-gray background (28.2 cd/m^2^). We manipulated the location of the arrow cue so that it was presented in the center of a randomly determined square on the grid, which corresponded to the locations and probabilities described in the previous section. The arrow cue was always a single arrow head. Participants completed eight blocks of 15 trials, preceded by one practice block.

### Results and discussion

#### Task performance

On average, participants were correct on 90.6 % (*SD* = 6.04) of trials. A repeated measures ANOVA showed no significant effect of trial length (five, six, or seven) on accuracy, *F*(2, 28) = 0.138, *MSE* = 2.570, *p* = .871. A planned linear contrast of sequence length revealed no significant linear trend in memory performance, *F*(1, 14) = 0.205, *p* = .658.

#### Reaction times

**Fig. 5:**
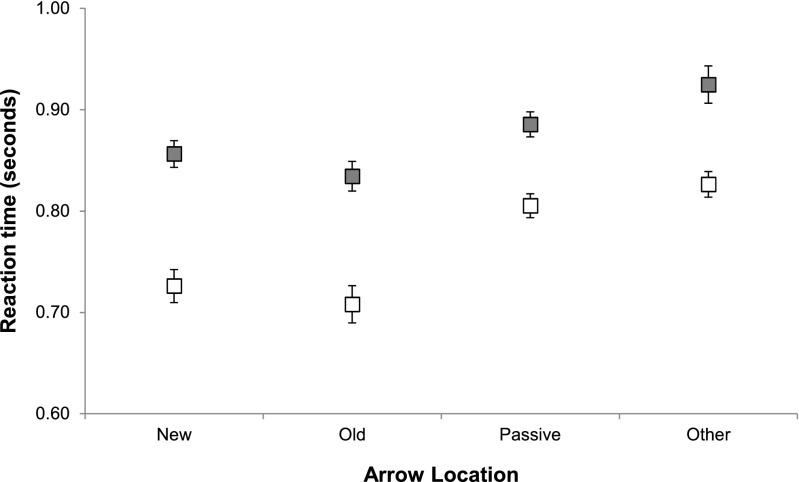
Average median reaction times (*N* = 15) in Experiment 2a for repetition (white) and switch (gray) updates, by the location of the arrow cue. Error bars indicate ±1 *SEM* corrected for within-subjects comparisons

A 2 (object switch condition) × 4 (arrow location) repeated measures ANOVA was performed on participants’ median RTs (see Fig. 5 for the RT data). A main effect of switch condition was observed, *F*(1, 14) = 36.202, *MSE* = .010, *p* < .001: Participants were faster to respond on repetition updates (*M* = 766 ms, *SD* = 237 ms) than on switch updates (*M* = 875 ms, *SD* = 282 ms). A main effect was also obtained for location, *F*(3, 42) = 28.400, *MSE* = .002, *p* < .001: Arrow cues appearing in the *old* location (*M* = 771 ms, *SD* = 264 ms) produced significantly faster RTs than arrow cues in the *passive* (*M* = 845 ms, *SD* = 265 ms, *p* < .001) and *other* (*M* = 876 ms, *SD* = 280 ms, *p* < .001) locations. Furthermore, arrow cues appearing in the *new* location (*M* = 791 ms, *SD* = 252 ms) produced significantly faster responses than arrows in the *passive* (*p* = .001) and *other* (*p* = .001) locations. Cues in the *passive* location were responded to marginally faster than cues in the *other* location (*p* = .071). The interaction of switch condition and arrow location did not reach significance, *F*(3, 42) = 1.323, *MSE* = .003, *p* = .28.

Comparing Fig. 5 to the predicted patterns in Fig. 3, the locations of objects kept in WM and the spatial orienting of perceptual attention were not completely independent of each another, contrary to the predictions of the independence hypothesis (see Fig. 3, panel ii-2). Participants were faster to respond when the arrow cue appeared in the location of the object to be updated (*old*) and when the arrow appeared in the *new* location for a target update, relative to cues appearing in the *passive* and *other* locations. The RT benefit for a spatial overlap between the location of the arrow cue and both the *old* and *new* locations of the to-be-updated object indicated that the overlap facilitated not only the refocusing of the object to be updated by the FoA in WM, but also the computation of that object’s new location. This corresponds to the predictions of the shared-map hypothesis (see Fig. 3, panel iii-2). As in Experiment 1, the data were again incompatible with the predictions of the dependence hypothesis, because the switch cost was not eliminated when the arrow cue was presented in a location incongruent with the current FoA in WM (see Fig. 3, panel i-2).

We argue that the benefit for spatial overlap arises because directing perceptual attention to a location facilitates shifting the FoA in WM to the same location. The reverse direction of causality—facilitation of processing of the arrow when it coincides with the current locus of the FoA in WM—can be ruled out as follows: If the location in the FoA in WM facilitated perceptual processing of an arrow that appeared in the same location, then on object switch trials, in which the passive object (i.e., the updated object of the previous step) was initially in the FoA in WM, an RT advantage should be observed for arrows in the passive location. No such advantage was observed.

Critically, the effects of arrow location (i.e., manipulating the orientation of perceptual attention) and of switch condition (i.e., manipulating the orientation of the FoA) were additive. This is as predicted by the third hypothesis outlined above (independent perceptual attention and FoA sharing a priority map), and contrary to the first and second hypotheses (that the FoA in WM is dependent on spatial attention, or that they are fully independent, respectively).

Experiment 2b replicated the same design with the 3 × 3 grid format.

## Experiment 2b

### Method

#### Participants

Seventeen undergraduate and postgraduate students, 18–31 of age, took part in the study, which was completed across two separate sessions, each lasting approximately an hour. Participants were reimbursed £15 for taking part. One participant did not return for the second session, and these data were thus excluded from the analysis.

#### Design and procedure

The general design and procedure were the same as in Experiment 2a, with the following exceptions: The grid was changed to a 3 × 3 grid extending 12.62° of visual angle; both testing sessions contained ten blocks of 15 trial sequences each; and the first test session included a five-trial practice block for participants to familiarize themselves with the task.

### Results and discussion

#### Task performance

On average, participants correctly identified the final locations of both objects on 82.3 % (*SD* = 10.24) of test trials. A repeated measures ANOVA showed a significant effect of trial length (five, six, or seven) on accuracy, *F*(2, 30) = 7.431, *MSE* = 15.915, *p* = .002. A planned linear contrast of sequence length revealed a significant linear trend in memory performance, *F*(1, 15) = 9.995, *p* = .006, with a decrease in performance for longer sequences.

#### Reaction times

**Fig. 6:**
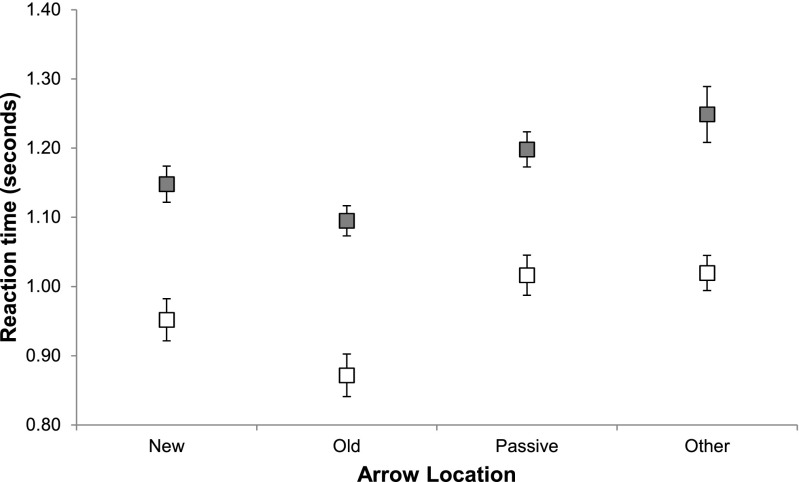
Average median reaction times (*N* = 16) in Experiment 2b for repetition (white) and switch (gray) updates, by the location of the arrow cue. Error bars indicate ±1 *SEM* corrected for within-subjects comparisons

A 2 (object switch condition) × 4 (arrow location) repeated measures ANOVA was performed on participants’ median RTs. Because the arrow appeared more frequently in task-irrelevant than in task-relevant locations, a random sample of these task-irrelevant updates (equivalent to the average number of updates contributing to the other locations) was selected for switch and repetition updates. Group averages of the individual median RTs for the different cue locations are plotted in Fig. 6. A main effect of switch condition was observed, *F*(1, 15) = 20.186, *MSE* = 0.068, *p* < .001; as in Experiment 1, a cost of switching was observed, with participants responding to repetition updates (*M* = 965 ms, *SD* = 316 ms) faster than to switch updates (*M* = 1,172, *SD* = 426 ms). A main effect was observed for arrow location, *F*(3, 45) = 26.985, *MSE* = .005, *p* < .001: Bonferroni-corrected post-hoc tests indicated that the RTs for updates in which the arrow cue appeared in the *old* location (*M* = 983 ms, *SD* = 358 ms) were significantly faster than updates in which the arrow appeared in the *new* location (*M* = 1,050 ms, *SD* = 386 ms, *p* = .005), the *passive* location (*M* = 1,107 ms, *SD* = 391 ms, *p* < .001), or the non-updating-related (*other*) locations (*M* = 1,134 ms, *SD* = 415 ms, *p* < .001). RTs for updates in which the arrow appeared in the *new* location were significantly faster than RTs for updates in which cues appeared either in *other* locations (*p* = .002) or in the *passive* location (*p* = .011). The interaction of update type and cue location did not reach significance, *F*(3, 45) = 1.01, *MSE* = .004, *p* = .399.

The replication in Experiment 2b of the additive benefits of object switching and location overlap observed in Experiment 2a further corroborates the shared-map hypothesis (iii) that we proposed: Perceptual–spatial attention and the FoA in WM are neither fully dependent nor completely independent; they can be oriented independently, but interact through a shared spatial priority map. While RTs to arrows appearing in the *new* location were faster than those to the *passive* and *other* locations (in line with predictions), they were slower than updates on which the arrow appeared in the *old* location (this difference was not statistically significant in Exp. 2a, though a similar trend can be observed in Fig. 5). We did not predict any difference between the old and new locations, only that an advantage would arise for both, but this difference does not contradict our account. In the order of the steps required to perform the update, discrimination of the arrow cue immediately precedes the selection of the object’s old location, whereas computation of the new location occurs later. Although activating the new location through the arrow cue facilitates the last step of the sequence, it could initially create competition with activating the old location in the preceding step. Therefore, on the basis of the assumption of competition between activation peaks in the priority map, activating the new location through the arrow cue should yield a smaller net benefit than activating the old location.

Previous studies examining the interplay between attention and WM have implicated eye movements as a strategic aid to performance—for example, as a rehearsal aid (e.g., Tremblay, Saint-Aubin, & Jalbert, 2006). This leads us to question whether eye movements were used strategically in our task. Could it be that the object switch cost, or the other effects that we observed, do not reflect the prioritization of an object in WM, but instead reflect participants using fixations to “mark” the locations of objects? Though attention shifts are thought to be obligatorily coupled to the target of an eye movement (Deubel & Schneider, 1996; Hoffman & Subramaniam, 1995), it is possible for participants to maintain fixation at one point and shift attention covertly to other parts of the visual scene (Posner, 1980). We have shown previously that making the appearance of each arrow cue contingent on participants returning their gaze to the center of the grid in between updates does not impact on the switch cost itself, nor on the patterns of eye movements that participants make while performing the update itself (Hedge & Leonards. 2013). Experiment 2c served to replicate this finding with the modified paradigm used in Experiments 2a and 2b.

## Experiment 2c

### Method

#### Participants

Sixteen undergraduate students, 18–31 years of age, took part in the study for course credit. The study was completed across two 1-h sessions.

#### Design and procedure

The stimuli and procedure were identical to those of Experiment 2b, with the modification that the appearance of the arrow cue was contingent upon participants fixating the central square of the grid. The interstimulus interval between updates was 460 ms followed by 40 ms (40 successive gaze samples) in which the eyetracker evaluated whether fixation fell in the center square of the grid presenting the next arrow. This ensured that participants had indeed fixated the center square before starting the next updating step, and that the interstimulus intervals were of comparable lengths in both experiments. After training, all participants returned eye gaze to the center square as requested.

Eye movements were recorded using an EyeLink 1000 desk-mounted eyetracker (SR Research, Ltd.). Eye movements were recorded on the basis of tracking the movement of the center of the pupil of the participant’s dominant eye at a rate of 1000 Hz, with a typical accuracy of 0.4° (or better) of visual angle. A nine-point grid calibration and validation were performed before beginning each block, and drift correction was performed in between trial sequences.

### Results and discussion

#### Task performance

On average, participants correctly identified the final locations of both objects on 83.35 % (*SD* = 12.50) of test trials. A repeated measures ANOVA showed a significant effect of trial length (five, six, or seven) on accuracy, *F*(2, 30) = 8.858, *MSE* = 15.27, *p* = .001. A planned linear contrast of sequence length revealed a significant linear trend in memory performance, *F*(1, 15) = 20.874, *p* < .001, with a decrease in performance for longer sequences.

#### Reaction times

**Fig. 7:**
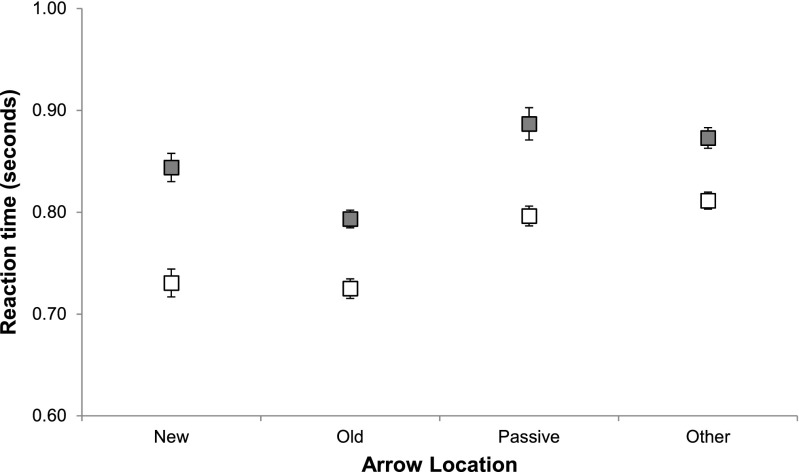
Average median reaction times (*N* = 16) in Experiment 2c for repetition (white) and switch (gray) updates, by the location of the arrow cue. Error bars indicate ±1 *SEM* corrected for within-subjects comparisons

Group averages of the individual median RTs for the different cue locations and object switch conditions are plotted in Fig. 7.

The pattern of RTs is similar to that observed in Experiments 2a and 2b. A main effect of switch condition was observed, *F*(1, 15) = 62.4, *MSE* = .004, *p* < .001, with participants responding to repetition updates (*M* = 766 ms, *SD* = 225 ms) faster than to switch updates (*M* = 849, *SD* = 250 ms). A main effect was observed for arrow location, *F*(3, 45) = 22.354, *MSE* = .002, *p* < .001: Bonferroni-corrected post-hoc tests indicated that the RTs for updates in which the arrow cue appeared in the *old* location (*M* = 759 ms, *SD* = 215 ms) were significantly faster than updates in which the arrow appeared in the *passive* location (*M* = 842 ms, *SD* = 248 ms, *p* < .001) and in non-updating-related (*other*) locations (*M* = 842 ms, *SD* = 257 ms, *p* < .001), and were marginally faster than RTs for the *new* location (*M* = 787 ms, *SD* = 240 ms, *p* = .067). RTs for updates in which the arrow appeared in the *new* location were significantly faster than RTs for updates in which cues appeared in the *passive* (*p* = .009) and in *other* (*p* = .001) locations. The interaction of update type and cue location did not reach significance, *F*(3, 45) = 2.14, *MSE* = .002, *p* = .108. Excluding updates on which the arrow cue appeared at fixation (the center square) did not change the pattern of updating latencies.^3^

In summary, the results from Experiment 2c indicate that the faster RTs observed on updates in which the arrow cue appears in the location of the object to be updated arises from the alignment of the target of perceptual information with the current FoA in WM. The alignment effect does not depend on the presentation of the arrow cue at fixation, nor on the use of fixations to maintain objects in priority in WM.

## General discussion

The aim of this work was to clarify the relationship between perceptual attention and the FoA in WM. Specifically, we examined whether perceptual attention is required to maintain an item in a prioritized state in WM. This question was motivated by recent observations that the FoA in WM interacts with perceptual attention by biasing perceptual inputs (Olivers & Eimer, 2011; Olivers et al., 2011). We used a spatial WM-updating task in which we could independently manipulate the orientation of attention in WM and the orientation of perceptual attention to either congruent or incongruent locations. We observed that the RT switch cost, which we assume reflects a benefit arising from a target object remaining in the FoA in WM, was not eliminated by drawing perceptual attention away from the current FoA to discriminate perceptual cues. This indicates that perceptual selective attention is not required to maintain an item prioritized in WM. In follow-up experiments, we observed faster RTs in the WM-updating task when perceptual cues aligned with updating-relevant locations. The effects of object switching and of alignment were additive, implying additive benefits of having the to-be-updated object in a perceptually attended location (i.e., aligned with the cue) and of having it in the FoA in WM (i.e., an object repetition trial). We conclude that selective attention in WM and selective attention in perception reflect independent mechanisms, but they interact in a shared representation of space, which we characterize as a spatial priority map (cf. Theeuwes et al., 2009).

### Potential objections

#### The role of arrow cue priming

One consideration concerns potentially confounding effects associated with the arrow cue. Repetition updates are associated not only with a repetition of the memory object to be updated but also with a repetition of the color of the arrow cue. Therefore, the object switch cost could arise from repetition priming facilitating perceptual processing of the cue. Cue priming can be separated from object switching by associating two cues to each object in WM. One experiment using this technique has shown that cue priming contributes about one third of the object switch cost (Gehring, Bryck, Jonides, Albin, & Badre, 2003). We have replicated the separate contributions of cue repetition and object repetition effects in a task similar to that presented here (unpublished data). The contribution of cue repetition priming to object switch costs therefore implies that in the present experiment we overestimated the size of the genuine cost of object switching. This overestimation, however, does not jeopardize our conclusion, because that conclusion does not depend on the size of the object switch cost.

In addition to cue color priming, our task also allowed a potential role of cue location repetition priming (i.e., faster RTs when the arrow cue appeared in the same grid square/location on two consecutive updates), which, though relatively infrequent, could have impacted the pattern of RTs that we observed. An analysis of these cue location repetition updates did indicate an RT benefit relative to nonrepetitions. However, a reanalysis of the data from our experiments with these updates excluded produced a near-identical pattern of results. This is to be expected because cue location repetitions were uncorrelated with the experimental variables.

#### Narrow or broad focus of attention in WM?

The interpretation of our findings is based on the proposal of a single-item FoA in WM: Whereas multiple items can be held in WM at any given time, one of these items is in a state of privileged accessibility to cognitive operations (Oberauer, 2002, 2003; McElree, 2001, 2006; McElree & Dosher, 1989). While there are converging lines of evidence for a narrow FoA in WM (for a review, see Oberauer & Hein, 2012), this has also been contested (Cowan, 2011; Gilchrist & Cowan, 2011). Therefore, we shall consider the compatibility of our findings with other models of WM next. First, some models conceptualize WM as a continuous resource that can be flexibly applied to representations (Bays & Husain, 2008; Wilken & Ma, 2004). In this conception, observers can strategically allocate resources to remembering a higher number of items at a cost in detail for the objects. If we assume that the object currently being updated requires more resources, and the switch cost reflects the reallocation of those resources, then our account is generally compatible with this class of models: What we called the “FoA” would then simply be the WM representation currently receiving an extra resource share.

A theory of WM assuming a broad FoA of around three or four items (Cowan, 1995, 1999) appears, at first glance, to be in conflict with our interpretation. By this account, both items in our updating paradigm are held in the FoA simultaneously and, by extension, the third slot could be occupied by a perceptual stimulus such as the arrow. However, for this account to be compatible with the RT costs of switching between objects, one would have to add the assumption that, within the broad FoA, one item is selected whereas the other is not, such that access to the currently selected item is faster than access to the not-selected item. The question that we addressed would then concern the relationship between perceptual attention and the mechanisms selecting one item in WM. Apart from replacing our term “working memory” with Cowan’s term “focus of attention,” and our term “focus of attention” with a novel mechanism for item selection, our account would be unchanged. In any case, we argue that our data are best accounted for by a model of WM in which a single item has privileged access to cognitive processing, and that perceptual selective attention does not underlie this privileged state in WM.

#### Unitary attentional system focusing multiple locations

One way to salvage a version of the dependence hypothesis would be by postulating a unitary attentional system responsible for the spatial selection of stimuli in the environment as well as locations in WM, with the ability to select two or more locations at the same time. Such a mechanism could select the to-be-updated object’s location in WM and the location of the arrow simultaneously. Two versions of this hypothesis could be considered:

1. *Unlimited capacity*: Perceptual attention can be allocated to multiple locations without a loss of performance. According to this account, a single (perceptual) attention mechanism could select the to-be-updated object and the arrow in different locations without cost. It would then follow that there should be no gain from aligning the location of the arrow and the location of the to-be-updated object, contrary to the results of Experiments 2a–2c.
2. *Limited capacity*: Perceptual attention can be shared among multiple locations, but doing so slows down the processing of information in each location. In many regards, this scenario matches our notion of a shared priority map with mutual competition between priority peaks. One remaining difference between our theory and a unitary attentional system selecting multiple locations is that our theory includes two independent selection mechanisms, whereas a unitary-attention model has only one selection mechanism that selects perceptual stimuli as well as objects in WM. Assuming a single selection mechanism that allocates limited attentional resources approximately optimally leads to the prediction that at the beginning of each update, the entire attentional resource is concentrated on the location(s) of the arrow(s), because there is no reason to keep the WM item updated on the previous step in the FoA. As a consequence, no object repetition benefit would occur whenever the arrow was presented in a location different from that of the last-updated object—this is the prediction that we derived from the dependence hypothesis above. The fact that we observed a substantial object repetition benefit that was additive with all manipulations of perceptual attention—the location of the arrows, as well as the difficulty of perceptual discrimination—shows that the selection of an object in WM is not undone by the subsequent selection of a perceptual stimulus. This persistence of the FoA in WM stands in contrast to the rapid and complete shift of perceptual–spatial attention: When perceptual–spatial attention shifts from location A to B, location A is rapidly deselected, and often even inhibited (Klein, 2000). The persistent selection of the last-updated item in WM shows that the selection mechanisms for visual stimuli and for items in WM operate independently.

### The interaction of perceptual attention and attention to information in WM

The present research is a direct extension of our earlier work in which we linked shifts in the FoA in WM to shifts in a spatial priority map (Hedge & Leonards, 2013), drawing upon models that propose a shared map between perceptual attention and WM (e.g., Theeuwes et al., 2009). We interpreted a correlation between eye movements and spatial shifts of attention in WM as being indicative of comparable processes of reorienting and selection between perceptual objects and the refocusing of memory items (Belopolsky & Theeuwes, 2009b, 2011). These processes are linked through a shared attention map, which feeds into a saccade map that guides eye movements. In this way, eye movements in the spatial-updating task tend to be directed toward a prioritized location in WM (i.e., the current FoA). However, as can be seen in Experiment 2c, we argue that the eye movements observed in our task are an epiphenomenon of shifts in priority in WM.

#### Spatial updating/switching and the priority map

In our previous work (Hedge & Leonards, 2013), we argued that the switch cost stems, in part, from resolving interference in a spatial attention map containing activations in multiple locations. In the case of an object switch update, on update *n*, the peak of activation begins in the *passive* location (the *new* location from update *n* – 1), and then must be shifted to the to-be-updated item’s location (the *old* location) before it can be updated to the *new* location. In contrast, on a repetition update, the peak of activation begins in the *old* location before being shifted to the *new* location. We propose that this difference in the number of peaks of activation over the course of the update (three on switch updates, two on repetition updates) is, at least in part, responsible for the slower RTs observed on switch updates. In the context of the present version of the task, in which the arrow cue varied in location, we extend this interpretation. When the cue appears in a location relevant for the updating process (i.e., the *old* or *new* location), the speed of updating is increased because perceptual attention adds to an already existing peak of activation in the priority map at a location relevant for updating. In contrast, when the arrow appears in another location, perceptual attention creates a peak of activation in a location of the map that is irrelevant for the updating task, potentially distracting/attracting attention (see also Lange, Starzynski, & Engbert, 2012).

**Fig. 8:**
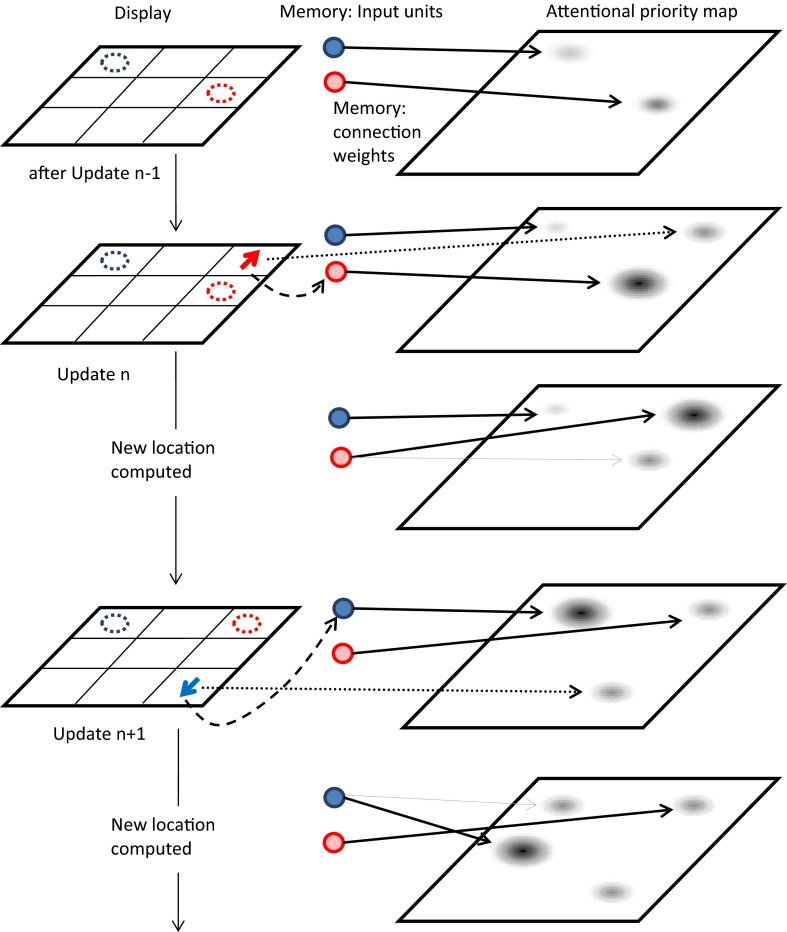
Framework of the mechanisms underlying performance in the spatial-updating/switching task. The flow of events is presented from top to bottom. On the left is the display, with the current memorized locations of both objects indicated by broken outlines. The right side shows the state of the working memory system, consisting of input units, representing the objects’ colors, and output units, representing their possible locations, organized into an attentional priority map, and the current bindings between input and output units (continuous arrows). The constellation on top represents the state following update *n* – 1, in which the red object was updated. Below it, a repetition update (*n*) illustrates the presentation of the red arrow stimulus in the *new* location. The peak of activity in the priority map shifts from the *old* location of the red object to the *new* location, and the binding between the “red” input unit is updated to the new location. A switch update from the red to the blue object is presented in update *n* + 1, to illustrate the shift in the attentional map from the *passive* location of the red object to the *old* location of the blue object, with the blue arrow cue appearing in an *other* location. The dotted line represents the effect of the arrow stimulus on the attentional map that is mediated through perceptual attention to the arrow’s location; the broken line represents the effect of the arrow stimulus on the map that is mediated through attention in WM to the object identified by the arrow’s color

Figure 8 schematically illustrates the mechanisms of perceptual attention and attention in WM, as they apply to our spatial-updating task. On a given updating step *n*, the arrow stimulus leads to modulation of the priority profile in the attentional priority map through two routes, one mediated through perceptual attention processes, the other mediated through attentional processes in WM. The perceptual attention process results in prioritization of the location of the arrow to facilitate its perceptual analysis. As a consequence, the location of the arrow receives an activation peak in the priority map. The attention processes in WM start with the activation of a color representation by the arrow’s color, which serves as a retrieval cue for the corresponding object’s location in the FoA of WM. Retrieval of the current location of the to-be-updated object relies on a matrix of bindings between colors and locations (Oberauer, Souza, Druey, & Gade, 2013). In the example in Fig. 8, on update *n*, the red color unit is bound to the middle-right location, because this is the current location of the red object in WM. As a consequence, the middle-right location receives a peak in the priority map. To ensure that the following processing steps—computing the *new* location of the red object according to the arrow’s direction—receives sufficiently unambiguous information about the current (*old*) location of that object as input, this peak must exceed all other peaks in the priority map. Besides the peak reflecting the location of the arrow cue, another peak is shown in the location of the other, currently not relevant object. This peak is higher on switch trials (the top-right location in update *n* + 1)—in which the other object has just been the relevant one—than on repetition trials.

The assumptions underlying this framework explain the results of our experiments as follows: The completion time for each updating step is influenced by the amount of conflict in the priority map between the relevant priority peak of activation—initially in each updating step, the old location of the to-be-updated object, later its new location—and other peaks of activation, such as the peak created by the arrow location and the peak representing the other object’s location. A high peak of activation in the *old* location facilitates rapid retrieval of that location as the starting point of the updating operation, and subsequently, a high peak in the *new* location facilitates computation of the new location of the object to be updated. High peaks of activation in the passive object’s location, or in the location of the arrow, slow down updating by competing with the relevant location (e.g., through lateral inhibition between peaks in different locations of the priority map). After completion of updating step *n* – 1, the new location to which the object has been shifted in that step remains slightly prioritized in the activation map.^4^ In repeat updates (update *n*), this location matches the *old* location. Therefore, the residual peak carrying over from step *n* – 1 adds to the activation peak generated in the *old* location of the to-be-updated object on trial *n*. In a switch update (*n* + 1), the residual peak carrying over from trial *n* does not match the *old* location of trial *n* + 1, and therefore competes with the new peak in the *old* location. This explains the object-switch cost. In addition, the location of the arrow generates an activation peak in the priority map, which adds to the peak in the relevant *old* or *new* location when the arrow is presented in one of these locations (update *n*), but competes with it when it is presented elsewhere (update *n* + 1). This explains the effect of arrow-cue location. The object switch effect and the arrow-cue effect are *additive* because the residual peak carrying over from the previous updating step and the peak generated through perceptual attention at the location of the arrow are generated independently.

#### Converging evidence

The proposal of a shared spatial map between WM and perceptual attention is in line with an increasing amount of behavioral and neuropsychological work (Bisley & Goldberg, 2010; Kuo, Rao, Lepsien & Nobre, 2009; Nobre et al., 2004; Theeuwes et al., 2009). Furthermore, the behavioral work on which Theeuwes et al.’s (2009) framework is based indicates that the sudden onset of an irrelevant perceptual item during a retention interval can cause the memorized location to be shifted toward the distracting item (Van der Stigchel, Merten, Meeter, & Theeuwes, 2007). Moreover, saccades to a perceptual target curve away from memorized locations (Theeuwes, Olivers, & Chizk, 2005), but not from a previously presented item that does not need to be retained, indicating a common representation of space that is used in perceptual processes and in WM processes. Thus, our conclusions converge with the conclusions from previous research on the interaction of perceptual attention and WM.

We could interpret the capture of attention in visual search by an irrelevant distractor item in WM (Olivers & Eimer, 2011; Olivers et al., 2011) within a similar framework: A match between a distractor in the search display and the item in the FoA boosts the activity of the distractor’s location in the priority map, thereby slowing down visual search, and potentially drawing eye movements toward it. Such attentional-capture findings have been interpreted as evidence for overlap between WM and attention, and here we specify the nature of this overlap: Attention in WM and in perception share a priority map—but their foci can be oriented independently to different locations in that map. Indeed, this separation between the content of the FoA in WM and selection of perceptual information is important for efficient processing in many visual tasks. For example, it would be inefficient if, when performing visual search, the memorized target was displaced from the FoA by each distracting visual stimulus that was evaluated.

Crucially, our framework accounts for several observations in the literature that are incompatible with a complete overlap between the mechanisms of perceptual attention and of attention in WM. First, as we noted in the introduction, when one item in WM is prioritized, the memory advantage of that item is robust to subsequent shifts of visual attention (Hollingworth & Maxcey-Richard, 2013; Maxcey-Richard & Hollingworth, 2013; Rerko et al., 2014).

Second, when performing a visual search task, a spatial WM load makes search performance less efficient, whereas a nonspatial visual WM load does not (Woodman & Luck, 2004; Woodman, Vogel, & Luck, 2001). Woodman and colleagues explained these results by assuming a spatial reference frame shared between WM and perceptual attention—indeed, this proposal mirrors our priority map account. Within our framework, when the memory task lacks a spatial element, it does not produce activity in the spatial priority map; thus, interference with the perceptual–spatial task is minimal.

Third, dual-task interference was smaller between a visual WM task and a task requiring perceptual attention to multiple items (i.e., multiple-object tracking) than between two WM tasks or between two object-tracking tasks (Fougnie & Marois, 2006). In other words, WM load and perceptual-tracking tasks draw upon some shared mechanism, but their capacities for representations can be separated. When object tracking and WM for visual objects in space share a spatial priority map, some degree of interference between those tasks is to be expected. At the same time, the visual attentional selection mechanisms required for object tracking can operate separately from the FoA in WM, implying that object tracking and visual WM do not have to share all of their mechanisms.

## Footnotes

1 It is not necessary to assume a particular capacity limit for perceptual attention here, but we assumed that perceptual attention is fully focused on the arrow cue, because at the onset of a new arrow cue, that arrow is the only stimulus that requires attention. We consider versions of the dependence hypothesis that would allow for splitting attention over multiple locations in the General Discussion.

2 For an account of how lateral intraparietal area (LIP), the region associated with the priority map according to Theeuwes et al. (2009), could perform such a function, see Bisley and Goldberg (2010).

3 One concern about the present experiments might be that, because we did not control eye fixations, the effects on RTs might merely reflect confounds of the independent variables with eye movement demands or constraints. There is no reason to assume such a confound. Experiment 2c and our previous work (Hedge & Leonards, 2013) showed that the RT switch cost arises independently of eye movement constraints. Furthermore, the cue location categories in our analysis were defined relative to the objects in WM; therefore, no physical features distinguishing these conditions could produce differences in the demands on eye movements. The results of a follow-up analysis of the data from Experiment 2c confirmed this. We repeated our RT analysis while additionally separating data on the basis of the location of the first saccade (*old*, *new*, *passive*, or *other*). This analysis replicated the main effects of update type and cue location (all *p*s < .001) on updating latencies, and no significant effects or interactions involved the location of the first saccade (all *p*s > .3).

4 Experiments and computational modeling work on the object switch cost have shown that the small peak of the previously updated object carrying over from trial *n* – 1 does not arise directly from incomplete erasure of that peak in the priority map. Rather, it arises from residual activation of the color representation in the memory input unit activated in trial *n* – 1, which re-creates a peak in the location bound to it (Oberauer et al., 2013).
